# Medial patellofemoral ligament is a part of the vastus medialis obliquus and vastus intermedius aponeuroses attaching to the medial epicondyle

**DOI:** 10.1007/s00167-022-06984-7

**Published:** 2022-05-06

**Authors:** Suthasinee Tharnmanularp, Akimoto Nimura, Masahiro Tsutsumi, Mio Norose, Sachiyuki Tsukada, Keiichi Akita

**Affiliations:** 1 Department of Clinical Anatomy, Graduate School of Medical and Dental Sciences, Tokyo Medical and Dental University, Tokyo, Japan; 2 Department of Functional Joint Anatomy, Graduate School of Medical and Dental Sciences, Tokyo Medical and Dental University, 1-5-45 Yushima, Bunkyo-ku, Tokyo, 113-8519 Japan; 3 Inclusive Medical Science Research Institute, Morinomiya University of Medical Sciences, Osaka, Japan

**Keywords:** Medial patellofemoral ligament, Vastus medialis obliquus, Vastus intermedius, Aponeurosis, Joint capsule, Adductor tubercle, Medial epicondyle, Cortical bone thickness

## Abstract

**Purpose:**

This study aimed to investigate the bony surface characteristic of the femoral attachment of the medial patellofemoral ligament (MPFL) and the correlation between the relevant layered structures, including muscular aponeurosis and the joint capsule, which contribute to patellofemoral joint (PFJ) stability.

**Methods:**

The morphology of the medial aspect of the medial condyle using micro-computed tomography and analysed cortical bone thickening in 24 knees was observed. For the macroscopic and histological analyses, 21 and 3 knees were allocated, respectively. The Kruskal–Wallis one-way analysis of variance test with Dunn post hoc testing was performed for statistical analysis.

**Results:**

At the level of the adductor tubercle, there were no significant differences in cortical bone thickness. At the level of the medial epicondyle (MEC), cortical bone thickness was considerably greater than that in other areas of the medial condyle (mean ± standard deviation, 0.60 ± 0.20 mm; *p* < 0.0001). Macroscopic analysis revealed that the deep aponeurosis of the vastus medialis obliquus and the tendinous arch of the vastus intermedius distally formed the composite membrane and adjoined to the joint capsule to firmly attach to MEC, which was located at 41.3 ± 5.7 mm posterior and 14.2 ± 3.1 mm superior to the joint cartilage. Histological analysis showed a composite membrane and adjoining capsule attached to MEC via fibrocartilage.

**Conclusion:**

MPFL could be interpreted as part of the deep aponeurosis of the vastus medialis obliquus (VMO) and the tendinous arch of the vastus intermedius, which combined with the joint capsule to attach to MEC. The cortical bone thickening indicated that the tensile stresses were loaded on MEC in aged cadavers. Involvement of VMO and vastus intermedius aponeuroses in restored graft of MPFL could utilise the dynamic stability of surrounding muscles to mimic a native structure.

## Introduction

Medial patellofemoral ligament (MPFL) has been commonly reconstructed to treat chronic lateral patellar instability [15, 20, 45]. However, there is currently no consensus on the method of MPFL reconstruction and rehabilitation that best restores natural function. Precise anatomical understanding of the MPFL is essential for managing the patellofemoral joint (PFJ) stability, especially since the femoral attachment sites have been demonstrated at various positions on medial femoral condyle in different studies [1, 45]. Histologically, the arrangements and boundaries of ligaments, tendons, and aponeuroses are vague [35]. Better anatomical understanding of these surrounding structures may help elucidate the mechanisms of the PFJ stability and further clarify the definition of the ligaments [2, 11, 41, 43].

Previously, high stresses transmitted through dense connective tissues, such as tendons, ligaments, and aponeuroses, have been shown to influence the morphology and cortical bone thickness at the attachment site [11, 25, 27, 37, 39, 40]. To date, the morphology of the medial condyle in relation to the tensile stress involve in the PFJ stability has been rarely discussed.

The aim of this study was to investigate the bony surface characteristic of the femoral attachment and the correlation between the relevant layered structures, including the muscular aponeurosis and joint capsule, in reference to the MPFL attachment. Considering the clinical implications, the involvement of surrounding aponeuroses in restored graft of MPFL could utilise the dynamic stability of muscles to mimic the structure of a native knee. It was hypothesised that the medial aspect of the medial condyle had a particular characteristic corresponding to the surrounding fibrous structures, which would contribute to PFJ stability, and after separating the adjoining vastus medialis obliquus (VMO), vastus intermedius aponeurosis, and joint capsule, the remaining part of these fibrous structures was assumed to be the MPFL.

## Materials and methods

### Cadaveric specimen preparations and micro-computed (micro-CT) tomography imaging

The ethical approvals was issued by the institutional review board of Tokyo Medical and Dental University (M2018-243-01). Twenty-five knees (11 pairs and 3 halves, including 13 right and 12 left knees) from 14 cadavers (six men, eight women; mean age and standard deviation, 84.6 ± 10.4 years) were obtained for this study. No specimens with a severe deformity of the knee joint or past histories of knee surgery were included. All the cadavers were donated to the Department of Anatomy of the Tokyo Medical and Dental University.

**Fig. 1:**
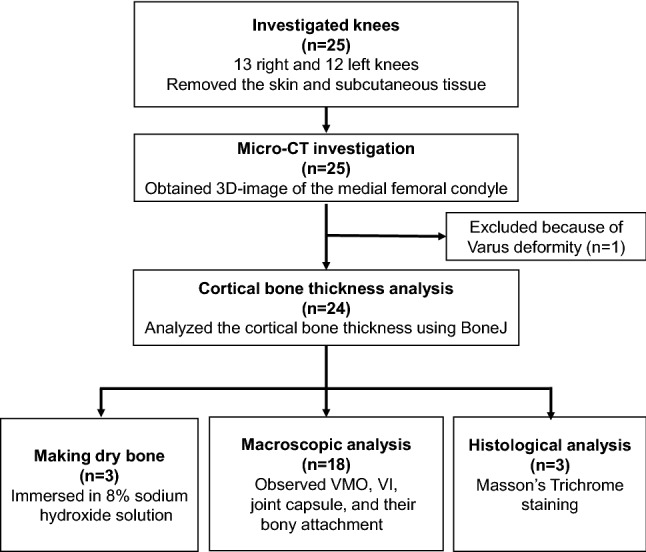
Flow diagram of the study enrolment process. *Micro-CT* micro-computed tomography, *VMO* vastus medialis obliquus, *VI* vastus intermedius

All specimens were fixed in 8% formalin and preserved in 30% ethanol. The medial regions of the knee were harvested by cutting at the distal one-third of the femur, tibial tuberosity, and midline of the sagittal plane using a diamond band pathology saw (EXAKT 312; EXKAKT Advanced Technologies GmbH, Norderstedt, Germany). After removing the skin and subcutaneous soft tissues, three-dimensional (3D) images of all medial halves of the knee using micro-CT (inspeXio SMX-100CT; Shimadzu Corp., Kyoto, Japan) with 200-µm resolution and ImageJ (version 1.52; National Institutes of Health, Bethesda, MD, USA) were obtained. One knee with a varus deformity on the 3D image was excluded. To confirm the correspondence between the 3D-CT image and actual bony surface without dissection artefacts, the soft tissues were removed in three specimens using an 8% sodium hydroxide solution (Wako Pure Chemical Industries, Osaka, Japan). In the remaining 21 specimens, 18 and 3 knees were randomly allocated to macroscopic and histological analyses, respectively (Fig. 1).

### Cortical bone thickness analysis of the medial condyle of the femur

To visualise the distribution of cortical bone thickening on the medial condyle of the femur, 8-bit images of 24 specimens obtained as described were transferred to ImageJ open-source image processing software (National Institute of Health, Bethesda, MD). The BoneJ plugin, an ImageJ extension for bone image analysis and published image processing algorithms, was used to define the thickness at a specific point by measuring the diameter of the largest sphere that fit within the structure of interest [6, 11, 16, 40]. The measurement was accurate for two-decimal places [10, 25]. The cortical bone thickness of the medial condyle was mapped in 3D images, in which brighter colours represented thicker points of the cortical bone.

To quantify the cortical bone thickness of the medial condyle, axial images at the middle levels of the adductor tubercle and medial epicondyle (MEC) were utilised. The locations of the measurements were divided into equal rectangles for the adductor tubercle (2.0 × 6.0 mm) and MEC (4.0 × 18.0 mm). The pilot trial was carried out to confirm that the rectangle fits the morphology of the cortical bone surface without overlapping boundaries in each measuring area. The rectangles were set parallel to the bony surfaces. The average cortical bone thickness of each rectangle was measured. The measurement was repeated twice in 24 knees randomly on different days using the same method. To determine the test–retest reliability, 48 measurements were compared between two examination days. All ICC scores were ≥ 0.92 (range 0.92–0.99).

### Macroscopic observation of the fibrous structures attaching to the medial epicondyle of the femur

After removing the skin and subcutaneous tissue, the sartorius, gracilis, and semitendinosus muscles were identified and reflected anteriorly. Next, the VMO was identified on the medial aspect of the knee. To identify the deep aponeurosis of the VMO, the VMO muscle portion was first removed. Then, the deep aponeurosis of the VMO was reflected anteriorly to reveal the vastus intermedius and its tendinous arch. The tendinous structures and joint capsule were detached en bloc from the medial condyle to clarify the relationships between the tendinous structures, joint capsule, and femoral attachment. Additionally, the location of the attachment on the femur’s medial aspect was measured using a non-digital Vernier calliper which allows measurement to one decimal. The measurements were taken twice in 18 knees on separate days using the same procedure. To validate the accuracy, 36 measurements were compared between two assessment days. All ICC scores were ≥ 0.98 (range 0.98–0.99).

### Histological analysis of the medial condyle of the femur

Three knees were randomly selected for histological analysis. Specimens were embedded in agar solution (22 g/L) and frozen at − 80 °C. Subsequently, specimens were cut horizontally into 5-mm-thick blocks using a band saw (WN-25-3; Nakajima Seisakusho, Osaka, Japan). Two blocks of axial slices were selected at the adductor tubercle and MEC levels. A 3D-CT image was used to validate the level. En bloc specimens were decalcified for 1 week in Plank-Rychlo solution (AlCl_3_:6H_2_O, 126.7 g/L; HCl, 85.0 mL/L; HCOOH, 50.0 mL/L) [33, 42, 43]. After decalcification, the blocks were dehydrated with a graded ethanol series, embedded in paraffin, and serially sectioned (thickness, 5 µm). Finally, staining was performed using the Manson trichrome staining protocol.

### Statistical analysis

The Kruskal–Wallis one-way analysis of variance test with Dunn post hoc testing was performed to compare the cortical bone thicknesses at measured locations in the axial slices at the adductor tubercle and MEC levels. Statistical significance was set at *p* values of < 0.05. To evaluate the test–retest reliability, a single observer randomly measured the specimens twice on different days in the same approach. The intraclass correlation coefficients (ICCs) were determined for each parameter. A score of > 0.75 was considered an excellent agreement. Data are presented as mean and standard deviation. Statistical analyses were performed using PASW Statistics 18 for Windows (SPSS Inc., Chicago, IL, USA).

To calculate the sample size for the cortical bone thickness analysis, the estimated cortical bone thickness from a previous relevant study that investigated the tibia and fibula was utilised [41]. Using the one-way analysis of variance, a 2-sided type I error of 0.05, 90% power, a minimum sample of 18 per area was required (nQuery Advisor^®^ Version 6.01; Statistical Solutions Ltd., Saugus, MA, USA). More samples were analysed to spare the number of specimens in case dropout during the study. The total number of investigated specimens in this study was 24.

## Results

### Bone morphological features of the medial condyle of the femur using micro-CT

**Fig. 2:**
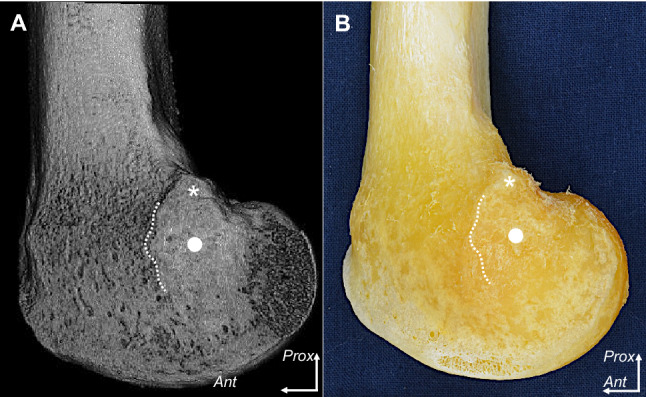
Morphology of the adductor tubercle and medial epicondyle. The medial aspect of the medial condyle of the right femur. The medial epicondyle (MEC; white circle) is located distal to the adductor tubercle (asterisk). The anterior margin of the MEC (dotted line) was remarkable in comparison with the posterior and inferior margins. **A** The medial condyle was examined using micro-computed tomography (micro-CT). **B** In the same specimen as **A**, soft tissues were chemically removed to assess correlations with the micro-CT image. *Ant*, anterior; *Prox*, proximal

Distal to the adductor tubercle, which was small and rough, the MEC was shaped as a wide shelving prominence (Fig. 2A). The proximal part of the MEC was continuous with the distal margin of the adductor tubercle. The anterior edge of the MEC was easily identifiable, whereas the posterior and inferior edges were unclear. These bony morphologies could be additionally confirmed as actual bone using chemical debridement (Fig. 2B).

**Fig. 3:**
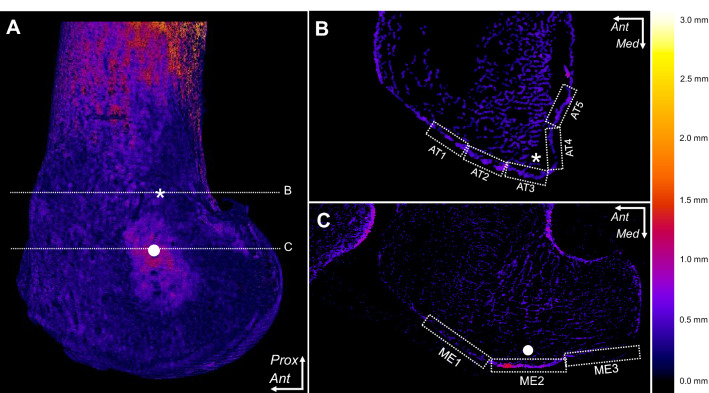
Evaluation of cortical bone thickening in the medial condyle of the femur. The cortical thickening maps on the right medial condyle were visualised after processing the micro-computed tomography (micro-CT) images. The thicker the cortical bone of the point, the brighter the colour of the point. **A** Three-dimensional image of the medial condyle surface. Levels of axial slices are shown as white dotted lines. **B** Axial image at the middle level of the adductor tubercle (asterisk). The mean cortical bone thickness was measured in each rectangle from the anterior to posterior part of the adductor tubercle (from AT1 to AT5). **C** Axial image at the level of the apex of the medial epicondyle (white circle). The mean cortical bone thickness was measured in each rectangle from the anterior to posterior part of the medial epicondyle (from ME1 to ME3). The cortical thickness distribution (mm) is shown as the spectrum from black to marine blue, violet, red, orange, yellow, and white. *Ant*, anterior; *Med*, medial; *Prox*, proximal

The cortical bone thickness measurement revealed that thickening was evident on the MEC, compared with the other areas of the medial condyle (Fig. 3). Data at the level of the adductor tubercle and MEC are shown in Table 1.

**Table 1:** The mean cortical bone thickness on the medial aspect of the medial femoral condyle

Location of Measurement	Mean ± SD (mm)
Adductor tubercle level	
Anterior most (AT1)	0.47 ± 0.10
Anterior to adductor tubercle (AT2)	0.49 ± 0.11
Anterior aspect of adductor tubercle (AT3)	0.43 ± 0.09
Posterior aspect of adductor tubercle (AT4)	0.45 ± 0.09
Posterior to adductor tubercle (AT5)	0.46 ± 0.12
Medial epicondyle level	
Anterior to medial epicondyle (ME1)	0.36 ± 0.08
Medial epicondyle (ME2)	0.60 ± 0.20*
Posterior to medial epicondyle (ME3)	0.36 ± 0.13

The area of the measurements is demonstrated in Fig. 2

*SD* standard deviation, *AT* adductor tubercle, *ME* medial epicondyle

*Highly statistically significant difference compared to the other areas (*p* value < 0.0001; the Kruskal–Wallis one-way analysis of variance test with Dunn post hoc test was conducted)

### Layered relationships between the fibrous structures attaching the medial epicondyle of the femur

**Fig. 4:**
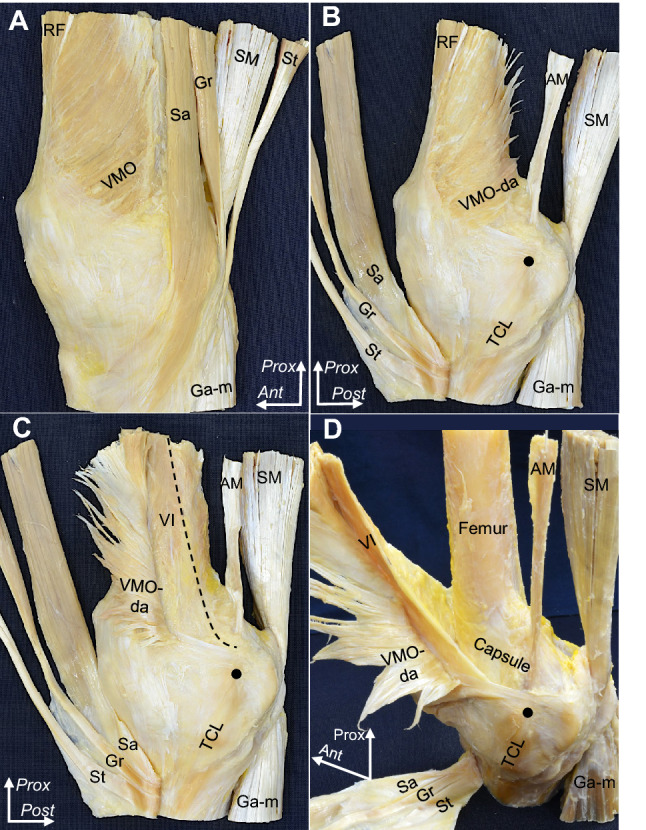
Layered relationships between the fibrous structures attached to the medial epicondyle. **A** The medial aspect of the right femur. The superficial fascia was removed, and the vastus medialis oblique (VMO) muscle is shown. **B** After removing the muscular portion of the VMO, the sartorius (Sa), gracilis (Gr), and semitendinosus (St) muscles are reflected to the anterior. The deep aponeurosis of the VMO (VMO-da) and posterior part of the composite membrane are exposed. **C** The VMO-da is reflected anteriorly. The tendinous arch of the vastus intermedius (VI) posterodistally continues to the composite membrane. The dashed line indicates the posterior margin of the tendinous arch of the VI. **D** The VI is reflected anteriorly. The composite membrane comprising the VMO-da and tendinous arch of the VI attaches to the MEC (black circle). AM, adductor magnus; Ga-m, medial head of the gastrocnemius; RF, rectus femoris; SM, semimembranosus; TCL, tibial collateral ligament; *Ant*, anterior; *Prox*, proximal

The deep aponeurosis of the VMO was observed to transition into the VMO tendon, which attached to the superomedial border of the patella (Fig. 4A, B). The tendinous arch of the vastus intermedius was exposed posteriorly to the VMO’s deep aponeurosis, which distally merged into the tendinous arch of the vastus intermedius to form the composite membrane (Fig. 4C). The joint capsule was underlaid deep into the composite membrane, which was distally intermingled with the joint capsule and firmly attached to the MEC (Fig. 4D).

**Fig. 5:**
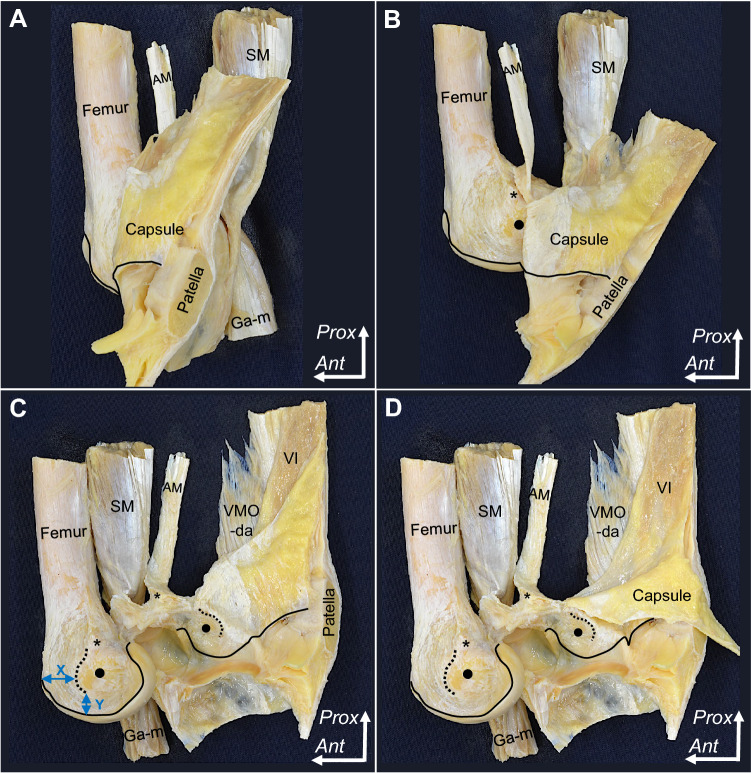
Attachment of the compositive membrane on the medial condyle of the femur. The tibia was removed from Fig. 3. **A** The medial half of the patellar bone and composite membrane, comprising the deep aponeurosis of the vastus medialis obliquus (VMO-da), tendinous arch of the vastus intermedius (VI), and joint capsule, are detached en bloc from the medial condyle of the femur and posteriorly reflected. Black solid lines indicate the proximal edge of the joint cartilage and corresponding part of the joint capsule. **B** The composite membrane is detached more posteriorly than **A**, and the adductor tubercle (asterisk) and medial epicondyle (MEC; black circle) are shown. **C** The composite membrane was detached more posteriorly than **B** to expose the firmly attached area to the MEC. Black dotted lines indicate the anterior margin of the firmly attached area of the composite membrane and the corresponding part of the membrane. The location of the firmly attached area on the MEC was measured from the medial (*X*) and proximal (*Y*) edges of the joint cartilage. **D** Distally reflecting the joint capsule, the connection between the joint capsule and tendinous arch of the VI is shown. AM, adductor magnus; Ga-m, medial head of the gastrocnemius; SM, semimembranosus; VI, vastus intermedius; *Ant*, anterior; *Prox*, proximal

Viewing from the intra-articular side, the deep part of the composite membrane was observed to distally merge with the joint capsule and firmly attach to the MEC (Fig. 5). The firmly attached area of the composite membrane and joint capsule are summarised in Table 2.

**Table 2:** The firmly attached area of the composite membrane and joint capsule on the medial aspect of the medial femoral condyle

Location	Mean ± SD (mm)
Posterior to the edge of the joint cartilage (*X*)	41.3 ± 5.6
Superior to the edge of the joint cartilage (*Y*)	14.2 ± 3.1

The area of the measurements is demonstrated in Fig. 5C

*SD* standard deviation

### Histological analyses of the fibrous structures on the medial condyle of the femur

**Fig. 6:**
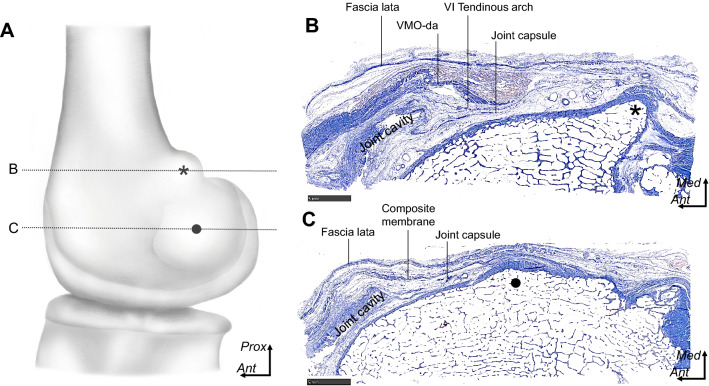
Histological analyses of the fibrous structures on the medial condyle of the femur. **A** Illustration of the medial aspect of the right medial condyle showing levels of the histological sections using Masson trichrome stain in **B** and **C** with dotted lines. **B** Axial section at the middle level of the adductor tubercle (asterisk). The deep aponeurosis of the vastus medialis obliquus (VMO-da), tendinous arch of the vastus intermedius (VI), and joint capsule are separated to form each layer. **C** The axial section at the middle level of the medial epicondyle (black circle in **A**). The composite membrane, comprising the VMO-da, tendinous arch of the VI, and joint capsule, is attached to the medial epicondyle via fibrocartilage. *Ant*, anterior; *Prox*, proximal; *Med*, medial. Scale, 5 mm

At the level of the adductor tubercle, the deep aponeurosis of the VMO was identified as a remarkable fibrous layer accompanied by a robust muscular portion (Fig. 6A, B). The joint capsule was identified as the innermost layer connecting the joint cavity. The tendinous arch of the vastus intermedius was observed as a thin fibrous layer interposed between the deep aponeurosis of the VMO and the joint capsule.

At the MEC level, the composite membrane adjoined the joint capsule and was attached to the MEC via fibrocartilage (Fig. 6A, C).

## Discussion

The most important findings of the present study were that the cortical bone thickness of the MEC was significantly greater than that of the other areas on the medial condyle, and the deep aponeurosis of the VMO and the tendinous arch of the vastus intermedius formed the composite membrane to firmly attach to the MEC via the fibrocartilage.

As previous reports, the bone is a highly adaptive structure under a mechanical stress load. Since the bone is thicker in areas that are subject to high stress, the distribution of cortical bone thickening could represent areas of high tensile stress, allowing the definition of functional attachment for fibrous structures [10, 25, 40]. The present study revealed that cortical bone thickening was limited in the MEC. This finding suggests that the tensile stress from the stabilising structures against lateral patellar translation is focussed on the MEC, regardless of the structure attached on it.

The MPFL is considered as a primary restrictor to maintain PFJ stability, whereas the medial patellotibial ligament (MPTL) and medial patellomeniscal ligament (MPML) are regarded as secondary stabilisers [7, 9, 17]. The MPFL has been described as a fan-like structure extending from the medial part of the femoral condyle to the superomedial edge of the patella [3, 19, 32, 45], associates with the VMO’s undersurface [13, 19, 26] and connects with the aponeurosis of the vastus intermedius [3, 23]. However, the layered relationship between the MPFL and surrounding structures, including the aponeurosis and joint capsule, has not been comprehensively discussed. In the current study, macroscopic and histological analyses showed that the deep aponeurosis of the VMO and tendinous arch of the vastus intermedius distally intermingle to form the composite membrane, which connects the medial side of the patella and the MEC. Based on the ambiguity regarding the definition of ligaments, the MPFL could be interpreted as part of the adjoining VMO and vastus intermedius aponeurosis if the fibrous part of the composite membrane was artificially separated. However, in vivo, the VMO and vastus intermedius seem to work together to restrain lateral translation of the patellar [28] rather than the independent static cord as assumed from the MPFL.

Recent studies have reported the MPFL’s femoral attachment [14, 30, 31, 46] at various sites, including at the adductor tubercle [24, 44], MEC [3, 5, 23], a point between the adductor tubercle and MEC [19, 29] and the gastrocnemius tubercle [1]. In short, the location of the MPFL’s attachment to the medial condyle remains inconclusive. Based on the results obtained in the present study, the previous inconsistency regarding MPFL femoral attachments could be explained as follows: although the MPFL could be part of the composite membrane, comprising the VMO and vastus intermedius aponeurosis, authors of previous anatomical studies may have assumed the MPFL to be a cord-like structure, artificially separate from the composite membrane, and thus defined the attachment locations differently. The actual composite membrane had a broader attachment to the MEC than the single point assumed by the cord-like MPFL model. In the current study, cortical bone thickening and fibrocartilaginous histology could validate the tensile stress on the MEC transferred by the composite membrane and joint capsule.

This study has several limitations. First, the sample size calculation for the cortical bone thickness analysis might be not be accurate, since previous data of the tibia and fibula and the one-way analysis of variance function were substituted. Second, the number of specimens used for the histological analysis was limited. Third, only the cadavers of older adults were used; therefore, the age was not matched with that of the patients with PFJ instability. Lastly, other bony morphologies were subject to the tensile stress in the relevant area, such as the gastrocnemius tubercle. Further studies are warranted to fully cover the thickness of the cortical bone on the medial side of the knee in relation to the soft tissue attachments. Consequently, we hope to lay the groundwork for subsequent anatomical and biomechanical studies with younger age population, or imaging of clinical cases of the medial part of the femoral condyle in the future.

This study emphasises certain clinical implications of the MPFL. First, considering the closely relationship between MPFL and VMO [28], the superior fibre of the MPFL graft meshed together with the VMO has been recommended during MPFL reconstruction [2, 12]. Based on anatomical findings, both the VMO and vastus intermedius were recommended to be involved in MPFL graft to utilise the dynamic stability of surrounding muscles to mimic the structure of a native knee. Second, since the VMO was thought to medially pull the patella through the MPFL by muscle contraction [18, 24], quadriceps strengthening has been the focus of conservative management strategies for PFJ stability [4, 8, 21, 34, 38]. The structural evidence for the effectiveness of quadriceps strengthening management could be validated in the present study. Third, femoral attachment malposition is a critical challenge when performing MPFL [18, 22, 24, 36]. The fact that the MEC corresponded to remarkable cortical bone thickening via fibrocartilaginous attachment of the composite membrane might support in determining the proper location of femoral tunnel fixation.

## Conclusion

The deep aponeurosis of the VMO and tendinous arch of the vastus intermedius distally intermingled to form a composite membrane, adjoining the joint capsule to attach to the MEC, which indicates that the MPFL was no more than a part of the adjacent VMO and vastus intermedius aponeurosis. The cortical bone thickening indicated that the tensile stresses were loaded on the MEC in aged cadavers. Involvement of VMO and vastus intermedius aponeuroses in restored graft of MPFL could utilise the dynamic stability of surrounding muscles to mimic a native structure.

## Abbreviations

PFJ: Patellofemoral joint
MPFL: Medial patellofemoral ligament
VMO: Vastus medialis obliquus
micro-CT: Micro-computed tomography
3D: Three-dimensional
MEC: Medial epicondyle
ICCs: Intraclass correlation coefficients
